# Phase Ib/II, open-label, dose-escalation study of LGX818, an oral selective BRAF inhibitor, in combination with MEK162, an oral MEK1/2 inhibitor, in patients with BRAF V600-dependent advanced solid tumors: preliminary results

**DOI:** 10.1186/1479-5876-12-S1-P5

**Published:** 2014-05-06

**Authors:** Richard Kefford, Ryan J Sullivan, Wilson H Miller, Elena M Elez, Daniel Tan, Kevin B Kim, Georgina V Long, Keith T Flaherty, David Tai, Simone Stutvoet, Heiko Maacke, Matt Whiley, Laure Moutouh-de Parseval, Josep Tabernero

**Affiliations:** 1 Melanoma Institute Australia, Westmead Institute for Cancer Research and Westmead Hospital, The University of Sydney, NSW, Sidney, Australia; 2 Massachusetts General Hospital Cancer Center, Boston, MA, United States; 3 Lady Davis Institute, Jewish General Hospital, McGill University, Montreal, Canada; 4 Vall d'Hebron Institute of Oncology, Barcelona, Spain; 5 National Cancer Centre Singapore, Singapore; 6 The University of Texas MD Anderson Cancer Center, Houston, TX, United States; 7 Novartis Pharma AG, Basel, Switzerland

## Background

Preclinical and clinical data suggest that combination of a BRAF and a MEK inhibitor (BRAFi, MEKi) may increase the efficacy over BRAFi monotherapy in BRAF-mutant metastatic melanoma and that addition of a MEKi may overcome or delay resistance to BRAFi.

## Materials and methods

This phase Ib/II study is evaluating the combination of LGX818, a potent, selective BRAF inhibitor, and MEK162, a selective MEK1/2 inhibitor, in patients with BRAF-mutant tumors (NCT01543698). The objectives for the phase Ib portion of the study were determination of the maximum tolerated dose (MTD) and/or recommended phase II dose (RP2D) for daily (qd) LGX818 + twice daily (bid) MEK162. Dose escalation was guided by a Bayesian logistic regression model with overdose control.

## Results

At the time of data cut-off (March 8, 2013), 30 patients were enrolled (melanoma, n =23 [9 BRAFi-naïve, 14 BRAFi-pretreated]; papillary thyroid cancer, n = 3 [2 BRAFi-naïve, 1 BRAFi-pretreated]; metastatic colorectal cancer, n = 4 [2 BRAFi-naïve, 2 BRAFi-pretreated]) and treated at the following dose levels (DLs) with LGX818 qd + MEK162 bid, respectively: 50 mg + 45 mg, 100 mg + 45 mg, 200 mg + 45 mg, 400 mg + 45 mg, 450 mg + 45 mg, and 600 mg + 45 mg. One dose limiting toxicity, grade 3 alanine aminotransferase elevation, was reported at the 600 mg + 45 mg DL. The MTD was not yet determined and two RP2Ds have been declared: 600 mg + 45 mg and 450 mg + 45 mg. Common adverse events (≥ 20%, all grades) suspected to be treatment related were nausea, diarrhea, fatigue, visual impairment, and headache. No events of fever, hyperkeratosis, or squamous cell carcinoma were observed. Among patients evaluable for response, complete response was observed in 1/9 (11%) BRAFi-naïve melanoma patients and partial responses were observed in 7/9 (78%) BRAFi-naive melanoma patients, 3/14 (21%) BRAFi-pretreated melanoma patients, and 2/3 (67%) thyroid cancer patients.

## Conclusions

Preliminary data indicate that the combination of LGX818 + MEK162 is active and demonstrates no substantial increases in adverse events compared to single-agent therapies. The absence of fever, hyperkeratosis, and squamous cell carcinoma suggest a distinct safety profile for this BRAFi/MEKi combination. Enrollment continues in the phase II portion of this study where the efficacy of LGX818 600 mg qd + MEK162 45 mg bid is being assessed.

